# The association of bone mineral density Z-score with the early postoperative remission and characteristics of bone mineral loss in patients with Cushing’s disease: a retrospective study

**DOI:** 10.3325/cmj.2022.63.578

**Published:** 2022-12

**Authors:** Emre Gezer, Zeynep Canturk, Alev Selek, Berrin Çetinarslan, Mehmet Sözen, Ozlem Elen, İhsan Anik, Savaş Ceylan

**Affiliations:** 1 Division of Endocrinology and Metabolism, Darica Farabi Training and Research Hospital, Kocaeli, Turkey; 2 Department of Endocrinology, Kocaeli University, Kocaeli, Turkey; 3 Department of Internal Medicine, Kocaeli University, Kocaeli, Turkey; 5 Department of Neurosurgery, Kocaeli University, Kocaeli, Turkey

## Abstract

**Aim:**

To investigate the association of bone mineral density (BMD) Z-scores with early-postoperative remission rate and clinical parameters in patients with Cushing’s disease (CD).

**Methods:**

We retrospectively evaluated the records of patients diagnosed with CD. After the exclusion of 230 patients, 87 CD patients were finally enrolled. BMD was determined by dual-energy x-ray absorptiometry (DXA) at the lumbar spine 1-4 (L_1–4_) and left femur. Early-postoperative remission was defined as a morning cortisol concentration on the first day after surgery of less than 5 μg/dL. The diagnosis of BMD “below the expected range for age” was defined as a Z-score≤-2.00 standard deviations.

**Results:**

DXA results were not significantly associated with early postoperative remission. They also did not significantly differ between eugonadal and menopausal groups. Preoperative morning cortisol significantly negatively but weakly correlated with Z-score of the total femur, while preoperative adrenocorticotropic hormone/cortisol ratio positively but weakly correlated with DXA results of L1-4.

**Conclusion:**

The severity of bone loss was not significantly related to the failure of transsphenoidal surgery for Cushing's disease.

Cushing’s disease (CD) is characterized by an overexpression of adrenocorticotropic hormone (ACTH) as a result of pituitary corticotroph adenoma. It leads to endogenous hypercortisolism, one of the most important causes of bone mineral loss (1). The prevalence of osteoporosis due to CD ranges from 34% to 50% (2-4). Glucocorticoid (GC) excess impairs bone metabolism through various direct and indirect mechanisms: increased bone resorption, reduced bone formation, impaired renal and intestinal calcium reabsorption, inhibition of gonadal steroids and growth hormone, and reduced muscle strength (5,6). The severity of CD increases the loss in bone mineral density.

Bone mineral density (BMD) is assessed with dual-energy x-ray absorptiometry (DXA) at the spine (anteroposterior and lateral) and hip (total femur and femoral neck). To detect the secondary causes of osteoporosis, such as CD, individual’s BMD is compared with that of an average person of the same sex and age. In this way, we obtain the number of standard deviations (SD) from age/sex-matched groups, ie, BMD Z-score (7). This study used Z-score as the main parameter to eliminate the effects of age, sex, and gonadal status on BMD.

The early outcome of pituitary surgery in patients with CD is affected by different factors: the surgeon’s experience, tumor extent and size, an accurate identification of the tumor during the operation or preoperatively on the MRI scan, preoperative ACTH levels, and the clinical severity of the disease (8-10). In addition, in patients with CD, ACTH and the severity of hypercortisolism influence the degree of bone mineral loss (3,11-13). Since the severity of hypercortisolism is related to both the early outcome of pituitary surgery and the degree of BMD loss, we postulated that BMD might be inversely related to early postoperative remission. Therefore, we investigated whether BMD Z-scores were associated with the early-postoperative remission rate and clinical parameters in patients with CD.

## Patients and methods

We retrospectively analyzed the electronic records (Nucleus and HUYE) of patients diagnosed with CD and operated on at the Pituitary Research Center of our institute between 2006 and 2020. CD was diagnosed with the following screening tests: midnight salivary cortisol, 24-hour urine free cortisol, a 2-mg low-dose dexamethasone suppression test, late-night serum cortisol, 8-mg high-dose dexamethasone suppression test, and corticotropin-releasing hormone (CRH) test or inferior petrosal sinus sampling, when needed. Overall, 317 patients were available for the analysis. Exclusion criteria were a lack of preoperative DXA results, preoperative MRI scan images, insulin-like growth factor 1 (IGF-1), cortisol, or ACTH levels; impaired IGF-1 levels (below or above the age-adjusted reference range); age less than 18 years; severe renal or hepatic failure; untreated/uncontrolled hypogonadism or hypo/hyperthyroidism; history of a disease or drug use that may cause secondary osteoporosis; lower spinal and hip fractures; and history of instrumentation use for stabilization of the lumbar vertebrae and hip. After the exclusion of 230 patients, there remained 87 eligible CD patients.

BMD was determined by DXA at the lumbar spine 1-4 (L_1–4_) and left femur (both femoral neck [F_N_] and total femur [F_T_]) using a GE Lunar Prodigy® 8743 densitometer (GE Healthcare, Machelen, Belgium). BMD values were expressed in grams per square centimeter. T-score and Z-score were expressed as standard deviation values, calculated through comparison with controls who were at peak BMD and persons matched for age and sex, respectively. BMD “below the expected range for age” was defined as a Z-score≤-2.00 SD (14).

Early-postoperative remission was defined as a morning cortisol concentration on the first day after surgery of less than 5 μg/dL (15-18). Pituitary adenoma size was evaluated with a MRI scan. The maximum tumor diameter and the presence of cavernous sinus invasion were noted. We also recorded the results of DXA scan, postoperative morning serum cortisol levels, patient demographics, preoperative morning cortisol and ACTH concentrations, gonadal status, and if the operation was primary or a re-operation. ACTH/cortisol ratio was calculated. The study was approved by the ethics committee of Kocaeli University, Faculty of Medicine.

### Statistical analysis

The normality of distribution was tested with the Kolmogorov-Smirnov and Shapiro-Wilk test. Continuous variables are expressed as mean ± standard deviation (SD) or median (25th-75th percentile). Categorical variables are summarized as counts (percentages). The groups were compared with independent samples *t* test or Mann-Whitney U test and one-way analysis of variance (ANOVA), as appropriate. The Tukey test was used for multiple comparisons. Correlations between continuous variables were determined with the Pearson and Spearman correlation analysis, and associations between categorical variables with the χ^2^ test. A two-sided *P* value <0.05 was considered statistically significant. Statistical analyses were performed with SPSS for Windows, Version 20.0. (IBM Corp, Armonk, NY, USA)

## Results

Demographics and clinical characteristics are summarized in Table 1. The prevalence of BMD “below the expected range for age” was 36.8% (n = 32) and that of normal BMD was 63.2% (n = 55) (Table 1).

**Table 1:** Demographic and clinical characteristics (n = 87)

Characteristic	n (%)
Age (years), median (min-max)	40 (18-73)
Sex	
male	14 (16.1)
female	73 (83.9)
Remission	
yes	48 (55.2)
no	39 (44.8)
Operation	
primary	77 (88.5)
secondary	10 (11.5)
Gonadal Status	
eugonadal	68 (78.2)
menopausal	19 (21.8)
Adenoma	
microadenoma	49 (56.3)
macroadenoma	38 (43.7)
Bone mineral density*	
below the expected range for age	32 (36.8)
normal	55 (63.2)

*Below the expected range for age and normal groups defined as Z-score≤-2 and>-2, respectively.

DXA results were not significantly associated with the early-postoperative remission (Table 2). They also did not significantly differ between eugonadal and menopausal groups (*P* > 0.05).

**Table 2:** The association of dual-energy x-ray absorptiometry results with remission and gonadal status*

		Remission			Gonadal status		
		yes^†^	no^†^	p^c^	eugonadal^†^	menopausal^†^	p^§^
L_1-4_	BMD	1.05 ± 0.17	1.07 ± 0.15	0.516	1.07 ± 0.16	1.01 ± 0.14	0.202
	T-score	-1.11 ± 1.45	-0.92 ± 1.27	0.512	-0.95 ± 1.40	-1.31 ± 1.24	0.306
	Z-score	-1.48 ± 1.39	-1.14 ± 1.21	0.232	-1.39 ± 1.40	-1.12 ± 0.96	0.338
F_N_	BMD	0.96 ± 0.18	0.96 ± 0.17	0.998	0.97 ± 0.18	0.90 ± 0.11	0.081
	T-score	-0.68 ± 1.20	-0.69 ± 1.12	0.970	-0.58 ± 1.23	-1.03 ± 0.79	0.135
	Z-score	-0.55 ± 1.23	-0.42 ± 1.12	0.604	-0.51 ± 1.27	-0.40 ± 0.78	0.713
F_T_	BMD	0.97 ± 0.17	0.95 ± 0.14	0.560	0.97 ± 0.17	0.94 ± 0.12	0.511
	T-score	-0.45(-1.38-0.78)^‡^	-0.30(-1.40-0.10)^‡^	0.494^¶^	-0.51 ± 1.25	-0.55 ± 0.93	0.891
	Z-score	-0.52 ± 1.31	-0.60 ± 1.01	0.745	-0.63 ± 1.23	-0.28 ± 0.95	0.252

*Abbreviations: BMD – bone mineral density; F_N_ – femoral neck; F_T_ – total femur; L_1-4_ – lumbar vertebrae 1-4.

†Data are expressed as mean ± standard deviation.

‡Data are expressed as median (25th-75th percentile).

§independent-samples *t* test.

^¶^Mann-Whitney U test.

There was a weak significant negative correlation between preoperative morning cortisol level and the Z-score of F_T_ (r = -0.276, *P* = 0.010, Table 3), while there was a weak positive correlation between preoperative morning ACTH/cortisol ratio and DXA results (BMD, T-scores, and Z-scores of L_1-4_: r = 0.280, *P* = 0.009; r = 0.258, *P* = 0.016; r = 0.257, *P* = 0.016, respectively). ACTH and adenoma size on MRI did not correlate with DXA results. Age did not correlate with preoperative morning cortisol, ACTH, or ACTH/cortisol (*P* > 0.05).

**Table 3:** Correlations between dual-energy x-ray absorptiometry results and preoperative morning cortisol, adrenocorticotropic hormone (ACTH), ACTH/cortisol ratio, and adenoma size*

		L_1-4_			F_N_			F_T_		
		BMD	T-score	Z-score	BMD	T-score	Z-score	BMD	T-score	Z-score
Cortisol	r	-0.115	-0.122	-0.177	-0.119	-0.104	-0.159	-0.219	-0.237	-0.276
	p†	0.289	0.261	0.100	0.272	0.336	0.827	0.041	0.027	0.010
ACTH	r	0.181	0.163	0.123	0.116	0.078	0.024	0.105	0.039	-0.21
	p†	0.094	0.130	0.255	0.285	0.473	0.827	0.335	0.720	0.845
ACTH/cortisol	r	0.280	0.258	0.257	0.199	0.156	0.126	0.249	0.206	0.160
	p†	0.009	0.016	0.016	0.065	0.150	0.244	0.020	0.055	0.139
Size of the adenoma	r	0.144	0.120	0.099	0.086	0.026	-0.001	0.109	0.060	0.002
	p†	0.182	0.270	0.361	0.430	0.808	0.993	0.316	0.584	0.983

*Abbreviations: BMD – bone mineral density; r – correlation coefficient; F_N_ – femoral neck; F_T_ – total femur; L_1-4_ – lumbar vertebrae 1-4.

†Spearman's Rho correlation test.

Z-scores of L_1-4_ were significantly lower than those of F_N_ and F_T_ (-1.33 ± 1.32 vs -0.49 ± 1.78, *P* < 0.001 and -1.33 ± 1.32 vs -0.55 ± 1.18, one-way ANOVA test with Tukey’s test *P* < 0.001, respectively, Table 4). Z-scores of F_N_ and F_T_ did not significantly differ (-0.49 ± 1.78 vs -0.55 ± 1.18, *P* = 0.934). Similarly, T-scores of L_1-4_ were significantly lower than T-scores of F_T_ (-1.03 ± 1.37 vs -0.52 ± 1.18, *P* = 0.021); however, there was no significant difference between T-scores of L_1-4_ and F_N_ and those of F_N_ and F_T_ (*P* = 0.158 and *P* = 0.672, respectively).

**Table 4:** Multiple comparisons between dual-energy x-ray absorptiometry results of each anatomic location*

	L_1-4_ mean±SD	F_N_ mean±SD	F_T_ mean±SD	F	p^†^
Z-score	-1.33 ± 1.32	-0.49 ± 1.78	-0.55 ± 1.18	**12.577**	**<0.001**
T-score	-1.03 ± 1.37	-0.68 ± 1.16	-0.52 ± 1.18	**3.788**	**0.024**
BMD	1.06 ± 0.16	0.96 ± 0.17	0.96 ± 0.16	**12.324**	**<0.001**

*Abbreviations: BMD – Bone mineral density; F_N_ – femoral neck; F_T_ – total femur, L_1-4_ – lumbar vertebrae 1-4, SD – standard deviation.

†one-way ANOVA test.

## Discussion

In our study, BMD Z-score, even though a parameter known to be affected by the severity of GC excess, was not related to the early postoperative remission rate in patients with CD. This is the first study to evaluate the indirect predictivity of Z-score for the early-postoperative remission in CD patients. Chronic hypercortisolism associated with CD negatively affects bone turnover, which causes osteoporosis, osteopenia, and BMD “below the expected range for age.” In our patients, osteoporosis prevalence was 16.1%, lower than the rate reported elsewhere (34% to 50%) (2,3). However, these studies generally had smaller sample sizes. Ohmori et al (2) reported a much higher prevalence of amenorrhea than we did (84.2% vs 21.8%). In the European Registry on Cushing’s syndrome, with the largest sample size to date, of 136 CD patients with DXA records (4), 22% and 12% were diagnosed with spine and hip osteoporosis, respectively.

A study by McKiernan et al (7), which included 18 674 patients, reported a significant inverse relationship between the presence of secondary causes of osteoporosis and Z-score. Similarly, Lewiecki et al (19) indicated that Z-score could be used for the diagnosis of osteoporosis caused by a secondary cause such as CD. Therefore, we decided to use Z-score as our primary variable, regardless of age, sex, and gonadal status. The prevalence of patients with Z-score below -2 SD was 36.8%, which was more than double the prevalence of osteoporosis. In a study by Sonino et al (10), patients with a severe clinical picture had significantly worse remission rates compared with patients without severe clinical picture. Additionally, in various studies DXA results negatively correlated with biochemical parameters indicating the severity of hypercortisolism (3,12,13,20). A hypothetical indirect relationship between DXA results and the early-postoperative remission derived from these findings was not supported by our study.

In accordance with multiple studies (12,21), we found no significant difference between DXA results and the gonadal status of CD patients. This finding shows endogenous hypercortisolism to be a more important determinant of bone metabolism than the gonadal status.

Our study showed that preoperative morning serum cortisol concentration significantly inversely, but weakly, correlated with the Z-score of F_T_. Similarly, another study demonstrated a significant negative association between serum cortisol and lumbar BMD (22). A recent study, which enrolled patients under long-term GC treatment, reported that cumulative GC dose negatively correlated with the Z-score of the total hip (23). Moreover, another study reported an inverse but non-significant correlation between presurgical morning cortisol level and BMD at both the lumbar spine and hip (12).

We also found a significant positive weak correlation between preoperative morning ACTH/cortisol ratio and DXA results of L_1-4_. Interestingly, the effect of ACTH alone on DXA results was non-significant, though the level of significance was higher for L_1-4_ than for F_N_ and F_T_. ACTH stimulates osteoblastic cell proliferation and increases collagen synthesis in osteoblasts (11). Similarly, it significantly positively correlates with the lumbar BMD (22). We found that excess GC had a deteriorating effect mainly on the total hip, while ACTH exerted a protective effect on the lumbar spine.

As one of the richest sites in the trabecular bone, the lumbar spine was most vulnerable to the effect of GC excess. Its high sensitivity to hypercortisolism was explained by higher bone turnover rate due to a greater volume/surface ratio. Kawamata et al (24) showed preoperative lumbar spine T-scores to be significantly lower than those of the femoral neck. The lumbar spine also sustained more severe bone loss than the hip (12). In our study, Z-scores and T-scores of L_1-4_ were significantly lower than those of F_N_ and F_T_.

The main limitation of our study was the fact that we did not assess body mass index and 25-hydroxyvitamin D levels, which were shown to be closely associated with bone structure and the osteoporosis development. Moreover, many patients were excluded from the study due to the absence of preoperative DXA scan.

In conclusion, our study found no significant relationship between the preoperative BMD Z-score and early-postoperative remission. To the best of our knowledge, this is the first study that investigated if the severity of bone loss predicted the failure of transsphenoidal surgery in CD. In addition, excess cortisol was negatively correlated with Z-score of F_T_ and ACTH/cortisol ratio was positively but weakly correlated with DXA results at the lumbar spine, which was the most adversely influenced bone site in CD. Bone properties were more affected by GC excess than by gonadal status.
